# Expression Quantitative Trait loci (QTL) in tumor adjacent normal breast tissue and breast tumor tissue

**DOI:** 10.1371/journal.pone.0170181

**Published:** 2017-02-02

**Authors:** Alejandro Quiroz-Zárate, Benjamin J. Harshfield, Rong Hu, Nick Knoblauch, Andrew H. Beck, Susan E. Hankinson, Vincent Carey, Rulla M. Tamimi, David J. Hunter, John Quackenbush, Aditi Hazra

**Affiliations:** 1 ArcBio, LLC, Cambridge, Massachusetts, United States of America; 2 Channing Division of Network Medicine, Department of Medicine, Brigham and Women’s Hospital and Harvard Medical School, Boston, Massachusetts, United States of America; 3 Department of Epidemiology, Harvard T.H. Chan School of Public Health, Boston, Massachusetts, United States of America; 4 Department of Pathology, Beth Israel Deaconess Medical Center and Harvard Medical School, Boston, Massachusetts, United States of America; 5 Division of Biostatistics and Epidemiology, School of Public Health and Health Sciences, University of Massachusetts, Amherst, Massachusetts, United States of America; 6 Program in Genetic Epidemiology and Statistical Genetics, Harvard T.H. Chan School of Public Health, Boston, Massachusetts, United States of America; 7 Broad Institute of MIT and Harvard, Cambridge, Massachusetts, United States of America; 8 Department of Biostatistics and Computational Biology and Center for Cancer Computational Biology, Dana-Farber Cancer Institute, Boston, Massachusetts, United States of America; 9 Department of Biostatistics, Harvard T.H. Chan School of Public Health, Boston, Massachusetts, United States of America; 10 Division of Preventive Medicine, Department of Medicine, Brigham and Women’s Hospital and Harvard Medical School, Boston, Massachusetts, United States of America; The University of Texas MD Anderson Cancer Center, UNITED STATES

## Abstract

We investigate 71 single nucleotide polymorphisms (SNPs) identified in meta-analytic studies of genome-wide association studies (GWAS) of breast cancer, the majority of which are located in intergenic or intronic regions. To explore regulatory impacts of these variants we conducted expression quantitative loci (eQTL) analyses on tissue samples from 376 invasive postmenopausal breast cancer cases in the Nurses’ Health Study (NHS) diagnosed from 1990–2004. Expression analysis was conducted on all formalin-fixed paraffin-embedded (FFPE) tissue samples (and on 264 adjacent normal samples) using the Affymetrix Human Transcriptome Array. Significance and ranking of associations between tumor receptor status and expression variation was preserved between NHS FFPE and TCGA fresh-frozen sample sets (Spearman r = 0.85, p<10^-10 for 17 of the 21 Oncotype DX recurrence signature genes). At an FDR threshold of 10%, we identified 27 trans-eQTLs associated with expression variation in 217 distinct genes. SNP-gene associations can be explored using an open-source interactive browser distributed in a Bioconductor package. Using a new a procedure for testing hypotheses relating SNP content to expression patterns in gene sets, defined as molecular function pathways, we find that loci on 6q14 and 6q25 affect various gene sets and molecular pathways (FDR < 10%). Although the ultimate biological interpretation of the GWAS-identified variants remains to be uncovered, this study validates the utility of expression analysis of this FFPE expression set for more detailed integrative analyses.

## Introduction

Genome-wide association studies (GWAS) of breast cancer have identified at least 71 risk alleles[1–3]. The majority of these single nucleotide polymorphisms (SNPs) are in intergenic or intronic regions. However, determining the target gene or biological pathway associated with these germline risk loci in breast tissue has remained a challenge. Identification of expression quantitative loci (eQTLs) associated with these SNPs may help us to better understand the mechanisms by which these risk variants influence breast cancer susceptibility. Previous eQTL studies evaluated a subset of these SNPs[4,5], using breast cancer cell lines, lymphoblastoid cell lines, reduction mammoplasty samples[6] or fresh frozen breast tissue from The Cancer Genome Atlas (TCGA)[7,8]. Although formalin fixed paraffin embedded (FFPE) tissue is the most common type of tumor tissue collected in the clinic, no comprehensive eQTL analyses of the 71 SNPs have been reported in FFPE breast tumor and tumor adjacent normal tissue specimens.

We validated the utility of the FFPE samples for expression analysis through comparative differential expression analysis of TCGA fresh-frozen samples. We then conducted eQTL analyses to test for associations between the 71 breast cancer risk SNPs and 26,004 array-defined transcript clusters in FFPE tissue from 376 tumor and 264 tumor adjacent normal breast specimens from postmenopausal breast cancer cases in the Nurses’ Health Study (NHS). In addition, we hypothesized that breast cancer GWAS loci are associated with regulation of biological pathways. To test this hypothesis, we developed a new method, functional quantitative trait loci (fQTL) analysis, and tested the association of the loci with 396 Molecular Functions in Gene Ontology (GO)[9].

## Results

**Fig 1:**
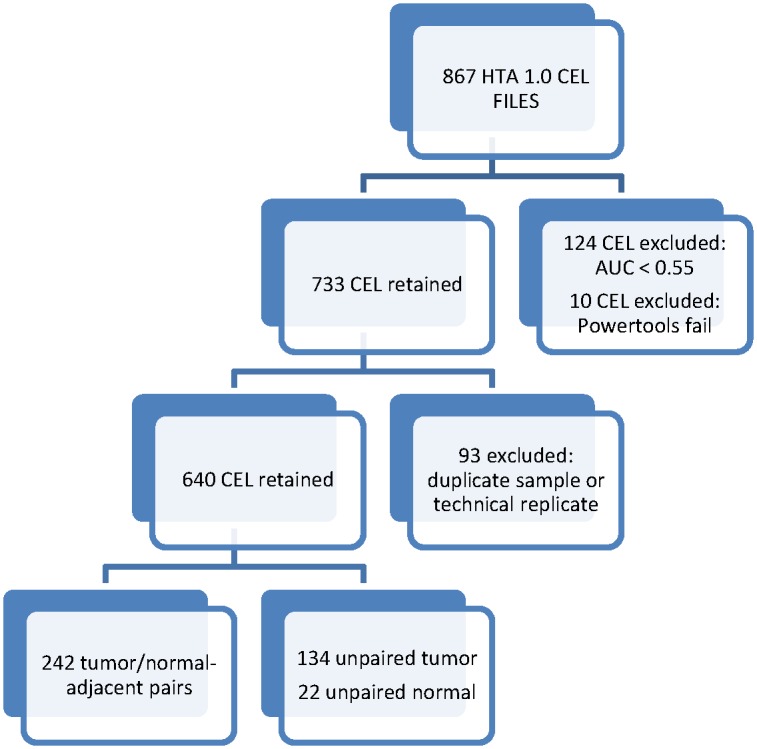
Derivation of expression samples from Nurses' Health Study participants for this analysis.

We analyzed QTL data from 376 postmenopausal invasive breast tumor specimens and 264 tumor adjacent normal specimens derived from an initial pool of 867 HTA 1.0 CEL files (Fig 1). The mean age at breast cancer diagnosis was 57 years and mean year of diagnosis was 1994. 262 (70%) of the breast cancer tumors with analyzable expression arrays were documented as ER positive (ER+). ER status in medical reports was used to update 44 (12%) specimens with missing data on estrogen receptor status from the expression assay.

### Adequacy of FFPE expression quantification

**Fig 2:**
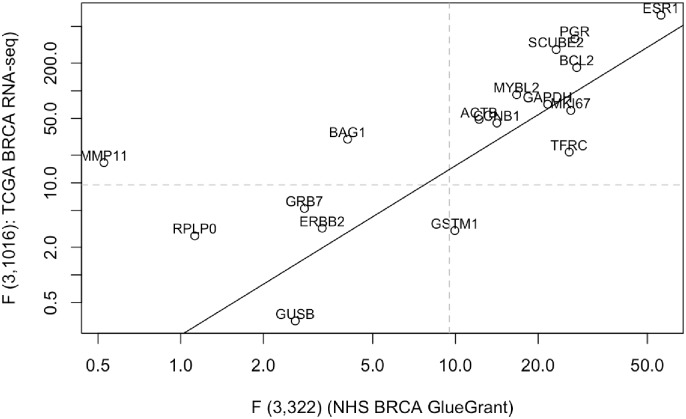
Concordance of F statistics derived from 1020 fresh-frozen TCGA and 326 FFPE NHS samples. Tests are for the 3 d.f. tests of common mean expression over ER+, ER-, PR+, PR- tumors, for 17 genes of the Oncotype Dx breast cancer expression signature. Spearman's r = 0.85, p < 10^-10. The guiding line is a robust regression fit with least trimmed squares.

It is understood that expression analysis of FFPE samples can be suboptimal owing to RNA degradation and other chemical and structural effects of fixation [10]. To build confidence in our expression quantifications, we acquired RNA-seq expression data on 1020 fresh-frozen breast cancer samples as archived at TCGA with date tag 2015-11-01. HUGO gene identifiers were matched between NHS and TCGA platforms for 9316 expression quantification targets. We focused on the capacity of expression measures to discriminate tumor hormone receptor status, as all samples were classified as estrogen receptor positive or negative (ER+ or ER-), and progesterone receptor positive or negative (PR+ or PR-). The 2 x 9316 F-statistics for the 3 d.f. tests of equal mean expression over ER+, ER-, PR+, PR- samples exhibited positive rank correlation (Spearman r = 0.43, p < 10^-10). A more focused assessment, based on 17 elements of the 21-gene Oncotype DX recurrence signature common to the NHS and TCGA platforms, yielded a Spearman's r = 0.85, p < 10^-10; see Fig 2. We conclude from this analysis that the RNA extracted from the NHS FFPE samples is suitable for differential expression analysis.

### Overall eQTL results

Seventy-one (71) breast cancer risk SNP were analyzed in this report; see S1 Table for their identifiers and information on genomic context. Separate analyses were performed for ER+, ER-, tumor adjacent normal, and contrast between tumor and tumor adjacent normal tissue. Test results were filtered to a maximum false discovery rate (FDR) of 10% per SNP, per tissue type. With this procedure we identified, using an additive genetic model based on called genotypes or imputed allelic dosage, a total of 27 trans-acting SNPs, exhibiting effects on expression of 369 unique Affymetrix Human Transcriptome Array (HTA) version 1.0 transcript clusters corresponding to 217 annotated genes. All but 10 significant associations involved SNPs and genes on different chromosomes; for the significant same-chromosome associations, the minimum SNP-gene separation was 6.4Mb. Consequently all associations are referred to as "trans-eQTL". Table 1 enumerates SNPs with significant trans-eQTL in terms of location, tissue-type analyzed, structural context of variants within genes, or epigenetic contexts of intergenic variants (as estimated with ChromHMM (11) on the HMEC cell line), the number of trans associations identified in different tissue types, minor allele frequency, and, when available, CADD score for variant deleteriousness, reported on the PHRED scale[11]. Findings in Table 1 are presented in the order: tumor vs. normal directly compared (5 SNP, column TUM/NOR), ER+ (4 SNP), ER- (7 SNP), multiple tissue findings (9 SNP), findings in normal tissue only (2 SNP). Table 1 condenses information to one record per SNP; full information on all significant associations is provided in S2 Table.

**Table 1:** Identification and annotation of 27 SNPs for which at least one SNP/tissue-specific analysis revealed an HTA 1.0 transcript cluster as a trans-eQTL target at FDR < = 10%.

dbSNP id	Chr	Addr. hg19	Tissues	Variant context	Transcript Clusters by Tissue				MAF	CADD
					ER+	ER-	NOR	TUM/NOR		
**rs4245739**	chr1	204518842	TUM/NOR	Intronic(*MDM4*)	0	0	0	*CD70*	0.258	NA
**rs2016394**	chr2	172972971	TUM/NOR	Quiescent/Low	0	0	0	Q9BU82	0.461	NA
**rs999737**	chr14	69034682	TUM/NOR	Intronic(*RAD51B*)	0	0	0	(UNANN)	0.207	4.49
**rs13329835**	chr16	80650805	TUM/NOR	Wk Rep PolyComb	0	0	0	*ABHD10*+1	0.237	1.26
**rs8100241**	chr19	17392894	TUM/NOR	Coding(*ANKLE1*)	0	0	0	*IL34*+1	0.496	NA
**rs10941679**	chr5	44706498	ER+	Enhancers	*CCDC51*	0	0	0	0.298	2.77
**rs11075995**	chr16	53855291	ER+	Intronic(*FTO*)	(UNANN)	0	0	0	0.231	1.25
**rs3760982**	chr19	44286513	ER+	Weak Tx	Q6ZRB7	0	0	0	0.484	1.73
**rs2284378**	chr20	32588095	ER+	Intronic(*RALY*)	*FAM123C*	0	0	0	0.314	NA
**rs17529111**	chr6	82128386	ER-	Weak Tx	0	24	0	0	0.251	NA
**rs2046210**	chr6	151948366	ER-	Quiescent/Low	0	(UNANN)	0	0	0.363	NA
**rs704010**	chr10	80841148	ER-	Intronic(*ZMIZ1*)	0	*HOXA6*	0	0	0.399	NA
**rs17356907**	chr12	96027759	ER-	Quiescent/Low	0	(UNANN)	0	0	0.293	1.1
**rs1292011**	chr12	115836522	ER-	Enhancers	0	*CDK8*	0	0	0.394	15.79
**rs3803662**	chr16	52586341	ER-	Enhancers	0	(UNANN)	0	0	0.283	0.008
**rs527616**	chr18	24337424	ER-	Enhancers	0	(UNANN)	0	0	0.365	16.26
**rs2380205**	chr10	5886734	ER+,ER-	Strong Tx	*TMEM126A*	(UNANN)	0	0	0.424	4.35
**rs11814448**	chr10	22315843	ER+,ER-	Enhancers	*KCTD13*	*FURIN*+1	0	0	0.018	0.09
**rs2588809**	chr14	68660428	ER+,ER-	Intronic(*RAD51B*)	(UNANN)	*IFITM1*	0	0	0.16	NA
**rs11571833**	chr13	32972626	(All)	Coding(*BRCA2*)	(UNANN)	186	25	*AADAT*	0.015	38.00
**rs10771399**	chr12	28155080	(All but ER+)	Wk Rep PolyComb	0	9	(UNANN)	*CCL3L3*	0.089	3.93
**rs132390**	chr22	29621477	(All but ER+)	Intronic(*EMID1*)	0	86	4	*GAGE12G*	0.027	7.53
**rs3757318**	chr6	151914113	NOR,TUM/NOR	Intronic(*CCDC170*)	0	0	4	5	0.085	NA
**rs12662670**	chr6	151918856	NOR,TUM/NOR	Intronic(*CCDC170*)	0	0	(UNANN)	*FGG*	0.09	NA
**rs9790517**	chr4	106084778	ER-,NOR	Intronic(*TET2*)	0	*HNRNPA1*	*ITSN2*	0	0.235	2.91
**rs4849887**	chr2	121245122	NOR	Quiescent/Low	0	0	(UNANN)	0	0.11	NA
**rs614367**	chr11	69328764	NOR	Quiescent/Low	0	0	*TNNC1*	0	0.169	0.27

Annotations for tissues and variant context are described in text. For each SNP, numeric entries in columns 6–9 are counts of transcript clusters identified as trans-associated in tissue-specific analysis. Minor allele frequency estimates were estimated for all women with available tumor samples. HUGO or UNIPROT symbols are provided for singleton hits; +1 denotes another hit present; use the interactive bceBrowse utility for further details. (UNANN) denotes unannotated HTA 1.0 transcript clusters.

**Fig 3:**
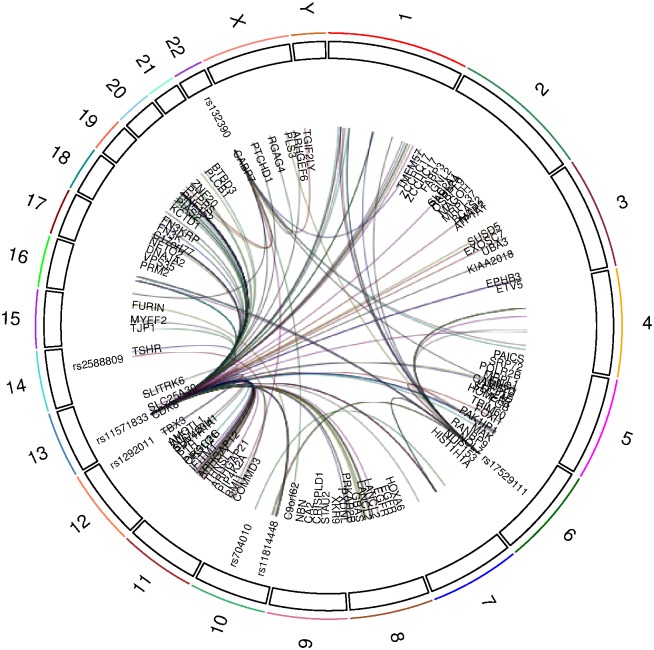
Circos visualization of SNP-gene pairs identified as trans-eQTL with expression measured in ER- tumor samples. Owing to label crowding, some SNP are not distinguished. Links lacking labels correspond to unannotated HTA 1.0 transcript clusters. Details on all significant associations are provided in the bceBrowse utility in the bceQTL package for Bioconductor.

We have created a browser-driven utility (bceBrowse, in Bioconductor package bceQTL, forthcoming, introductory video at https://www.youtube.com/watch?v=fJDXI5M7_mQ) that allows searching, sorting and plotting the FDR-filtered association results to facilitate tabulating and navigating of our data. A Circos display of trans-eQTL relationships for expression measured in ER- tumors is provided in Fig 3. Supplementary S1 and S2 Figs depict the relationships for findings in paired tumor/adjacent normal, and ER+ tumors.

**Fig 4:**
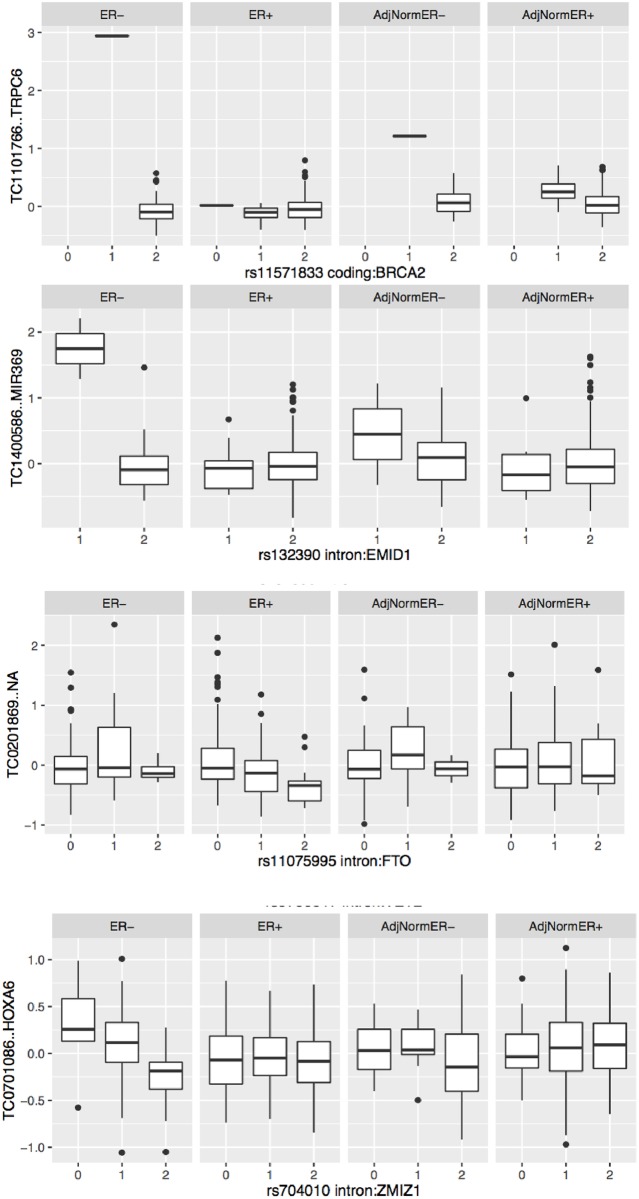
a-d. Trans-eQTL findings for SNPs in *BRCA2*, *EMID1*, *FTO*, *and ZMIZ1*. In Fig 4a, the coding SNP in *BRCA2* exhibits statistically significant association with expression of *TRPC6*, but the result is based on a single individual with a single copy of the rare allele. In Fig 4b, an intronic SNP in *FTO* shows association specific to ER+ cases with abundance of an HTA 1.0 probe that has yet to be annotated. In Fig 4d, an intronic SNP in *ZMIZ1* with tumor- and receptor-type specific effect on expression of *HOXA6*.

To illustrate variation in expression present in trans associations inferred in this study, the bceBrowse utility will display boxplots for selected SNP-gene pairs. Fig 4 provides examples of 4 trans-eQTL findings, each stratified by ER status and tissue type (tumor vs. adjacent normal).

### Functional QTLs

We tested SNPs for association with gene groups defined by the Molecular Function (MF) Gene Ontology terms to identify pathway-level associations for the 71 breast cancer loci. The associations presented in Table 2 demonstrate that a unit increase in the dosage of a SNP modifies the mean expression of the genes in a pathway-specific gene set categorized as the MFs in GO.

**Table 2:** Results of fQTL analysis applied to all breast tumor and adjacent normal samples.

Tissue source	dbSNP ID	Context	SNP position	Pathway (GO MF)	Risk beta	FDR
**Normal**						
	rs12662670	*CCDC170* (intronic)	6p25	Small GTPase binding	-4.3	0.048
ER+						
	rs6762644	*ITPR1* (intronic)	3p26.1	Phospholipase activity	4.3	0.075
ER-						
	rs2823093	intergenic	21q21.1	Oxidoreductase activity	4.52	0.03
	rs17529111	intergenic	6q14	Hydrolase activity	4.41	0.06
				Neuropeptide hormone activity	5.09	0.006
				Sialyltransferase activity	4.46	0.052
	rs17530068	intergenic	6q14	Hydrolase activity	4.39	0.066
				Sialyltransferase activity	4.53	0.039
				Nucleotide kinase activity	4.52	0.041

## Discussion

At a SNP-specific false discovery rate threshold of 10% in separate analyses of ER+, ER-, and tumor-adjacent normal paired samples, 27 of 71 meta-analytically identified breast cancer risk SNP exhibited association with mean expression of at least one HTA 1.0 transcript cluster. The total number of genes significantly associated with these SNPs in trans is 217. Five SNPs exhibited association with mean expression of gene sets defined using Gene Ontology molecular function categories; three of these fQTL did not show significant association with any transcript cluster in SNP-gene association testing. This analysis therefore distinguishes a total of 30 of 71 breast cancer risk SNPs as potentially acting through effects on gene expression. Tables 1 and 2 indicate that the majority of these SNPs are in intergenic or intronic regions.

Two of the breast cancer loci identified as trans-eQTL in this study involve DNA repair pathways. These are rs11571833 (nonsense mutation in *BRCA2*), and rs2588809 (intronic in *RAD51B*). The *BRCA2* variant, rs11571833, on exon 27, results in a premature stop codon *p*.Lys3326*, removing the last 92 amino acids at the C terminus of *BRCA2* and shown to be pathogenic in *in vitro* splicing assays[12] and associated with risk of breast, prostate and ovarian cancers[13,14]. The *BRCA2* CCOH terminus interacts with Rad51 and homozygous germ-line deletion of exon 27 disrupts homologous recombination-mediated DNA repair[15,16] and results in hypersensitivity to ionizing radiation and rapid senescence[17]. *RAD51B*, a member of the Rad51-like proteins, is involved in double-stranded break (DSB) repair and homologous recombination.

The trans-acting SNPs (Table 1) in *CCDC170* are located in the region on chromosome 6q25 in which a fusion event has been reported in breast cancer between the second exon of *ESR1α* to the sixth and seventh exon of *CCDC170[18]*. The rs12662670 eQTL and fQTL as well as the rs3757318 eQTL are located 272 to 327 kilobases away from the genomic location of the *ESR1-CCDC170* fusion in breast cancer cell lines and patient samples (Reference [18] and personal communication with Dr. Xiaosong Wang). Further studies[19] are warranted to assess if the QTLs in *CCDC170* are associated with the *ESR1-CCDC170* fusion in aggressive ER+ tumors. Variants on 6q25 associated with ER- tumors are located in four separate enhancer elements and are associated with reduced expression of *ESR1* and *CCDC170*.

Rs17529111, on chromosome 6 and 5’ to *FAM46A*, was identified as a trans-acting SNP and an fQTL in ER- tumor tissue. The variant was associated with an almost two-fold increased risk of triple-negative breast cancer among African American women[20]. Our eQTL and fQTL results for rs17529111 suggest that it may be a potential regulatory SNP in ER- breast tumors. Further bioinformatic and experimental analysis of these variants will be necessary to fully elucidate mechanisms of germ-line variant modulation of breast cancer risk.

We were unable to document any significant associations between the risk SNP analyzed in this report and expression of genes *in cis*. S1 Table includes information derived from GTEx [21] version 6 analyses of 183 *post mortem* normal breast tissue samples. Five SNPs, three intronic, one intergenic, one coding, are identified in GTEx as cis-eQTL in normal breast tissue. Denser genotyping of the FFPE samples from NHS could yield more insight into cis regulation processes affected by the SNP available in this analysis.

The strengths of our study are in providing direct insight into SNP associated transcriptome-wide and pathway-based perturbations in a large dataset of breast tumor tissue and paired tumor adjacent normal tissue with well-annotated histopathological data and lifestyle risk factor data. Our approach to integrate germline genetic risk alleles, somatic transcriptome data with risk factor data and functional annotation data may enhance the precision of eQTL analyses. In addition, the study provides validation for FFPE data quality for gene expression using fresh frozen breast tumor samples that were included in the TCGA and outlines the quality control best practices for FFPE tissue processing in gene expression analyses. Further, to enhance data sharing, we have developed a breast cancer eQTL browser (bceQTL).

This study has limitations. Large-scale post-menopausal breast cancer datasets with transcriptome data measured in breast tumor and tumor adjacent normal tissue are not available for independent external validation of *cis* and *trans* eQTLs. Our study was also limited by the available genomic annotation. No cis regulatory variants were identified using rigorous thresholds for statistical significance, although our identification of potentially regulatory SNPs were consistent with single-gene fine mapping studies and single-gene functional studies.[14,22–29] Further studies are needed to validate single SNP cis-eQTL analysis conducted using the TCGA data[3,7,30].

*Trans* analysis in TCGA data identified three risk loci, *ESR1*, *MYC*, *KLF4*, for which the target genes are significantly enriched for transcription factor motifs.[7] These data demonstrate that eQTL analyses offer insight on the breast cancer GWAS SNPs that are in introns or intergenic regions. Strength of the TCGA dataset is the sample size and ability to adjust for somatic alterations. However, inability to account for clinical and lifestyle risk is a weakness.

Another limitation is that the gene expression profiling from tumor cores may reflect heterogeneous profiling of tumor epithelial and stromal cells^33^. Laser capture microdissection to isolate specific cell types was not feasible considering the large sample size in this study. Further, data on neoadjuvant endocrine treatment, which may influence estrogen related genes, is not available in our dataset or in TCGA. Finally, our study is generalizable to Caucasian women. Diverse study populations and improved methodology to measure and analyze differential transcript isoforms, transcription factors in FFPE tissue may identify and validate additional eQTLs.[26] [31–33]

In summary, this large-scale study of paired breast tumor and tumor adjacent normal tissue demonstrates the adequacy of FFPE based gene expression studies for cis and trans eQTL analysis, and indicates that QTLs can be identified using FFPE issue with rigorous quality control metrics, including patient sample filtering, probe filtering, PCA to adjust for batch-to-batch variation in sample preparation (e.g. RNA extraction), and evaluation of subtype concordance by immunohistochemistry protein expression and HTA transcript cluster probe. Over 90% of patient specimens collected worldwide are FFPE. Therefore, QTL analysis in clinically relevant FFPE tissue that has been processed according to standard quality assurance protocols should be useful for detailed investigation of transcriptional effects of genomic variants. While this analysis indicates that several breast cancer risk SNP exhibit significant trans association with numerous genes, the minor allele frequencies of these SNP are relatively low, and the inferences on variant:expression association are driven by small numbers of individuals. Further confirmatory work with larger sample sizes and more intense bioinformatic investigation of unannotated HTA transcripts is warranted.

## Methods

### Study population

For any report of breast cancer, written permission was obtained from participants to review their medical records to confirm the diagnosis and to classify cancers as in situ or invasive, by histological type, size, stage of disease and presence or absence of metastases. The Human Subjects Committee at Partners Healthcare System and Brigham and Women’s Hospital in Boston, Massachusetts, have reviewed and approved this study.

The Nurses’ Health Study cohort was established in 1976 when 121,701 female US registered nurses ages 30–55 responded to a mailed questionnaire inquiring about risk factors for breast cancer. Study participants reported new breast cancer diagnoses on subsequent biennial questionnaires. Pathology reports were also reviewed to obtain information on estrogen receptor (ER), progesterone receptor (PR), and Human Epidermal growth factor Receptor 2 (HER2) status. Collection procedures for the breast cancer tissue blocks have been described in detail previously[34]. Participants complete a biennial questionnaire on dietary and lifestyle factors, reproductive factors, anthropomorphic measures, medication use, and health outcomes. Health outcomes include nonfatal incident diseases such as cancer. The follow-up rate in this cohort has been over 90%[35].

The Cancer Genetic Markers of Susceptibility (CGEMS) project (http://www.cgems.cancer.gov) is an NCI initiative to conduct genome-wide association studies (GWAS) to identify genes involved in breast and prostate cancer[36]. As a part of this project 1,145 cases were genotyped using the Illumina 550 array (data is publically available in dbGAP). For the current analysis, we identified invasive postmenopausal breast cancer cases diagnosed from 1990–2004 with GWAS data from the CGEMS project and sufficient RNA for expression profiling in breast tumor and tumor adjacent normal breast tissue.

Informed consent was obtained from all Nurses’ Health Study participants, including consent to collect and use blood samples and tissue specimens for genetic and genomic research[1,35]. This study was reviewed by the Internal Review Board and approved by the Human Subjects Committee at Partners Healthcare System and Brigham and Women's Hospital in Boston, Massachusetts.

### SNP selection

The 71 germline genetic variants used in the eQTL and fQTL analyses were the published loci from breast cancer GWAS and the U19/GAME-ON meta-analysis [1–3] (http://gameon.dfci.harvard.edu). Genotyped and imputed SNP dosages were obtained from the CGEMS study[1] breast cancer cases.

### Gene expression assay methods

RNA was extracted from multiple 1 or 1.5 mm cores (median number of cores was 5 in normal samples and 3 in tumor samples) taken from representative portions of tumor or adjacent normal from the FFPE blocks. The RNA extraction was performed using the Qiagen AllPrep RNA isolation kit. Total RNA was amplified using the NuGEN WT-Ovation FFPE System (NuGEN, San Carlos, CA). Total RNA was used to synthesize double-stranded complementary DNA (cDNA) utilizing a random priming method and then double-stranded cDNA was fragmented, labeled and hybridized to the Affymetrix Glue Grant Human Transcriptome Array (HTA 1.0) pre-release version from Affymetrix, Santa Clara, CA[37]. CDNA yield was measured in subset of samples prior to hybridization as a quality control measure. After hybridization and washing, processed slides were scanned with the Affymetrix GeneChip Scanner 3000 7G at the Dana-Farber Cancer Institute Microarrray Core Facility.

The Glue Grant Human Transcriptome Array was developed for high-throughput clinical studies, allowing for comprehensive examination of gene expression and genome-wide identification of alternative splicing as well as detection of coding SNPs and noncoding transcripts(23). The microarray can detect 34,834 lncRNAs and 39,224 transcript clusters (genes) curated from the most authoritative databases such as RefSeq and Ensembl as well as the literature. Repeat sequences and ncRNAs shorter than 200 bp were deleted. Each transcript was represented by 119 unique probes (on average) to improve statistical confidence. Each transcript was represented by a specific exon or splice junction probe that can accurately identify individual transcripts.

### Quality control analysis

The quality control procedures include patient sample filtering, probe filtering, PCA to adjust for batch-to-batch variation in sample preparation (e.g. RNA extraction), and evaluation of subtype concordance by immunohistochemistry and HTA probe. In Fig 1, we summarize the number of NHS participants at each stage of the study, from 774 eligible breast cancer specimens to the final sample size (639 specimens with gene expression and genotype data) included in the analysis. To ensure data quality, we conducted quality control analyses (correlation of four independent technical replicates across nine assay plates r≥0.93), biological data checks (probe concordance with markers measured by immunohistochemstry), as well as probe filtering and Principal Components Analysis (PCA) to correct for assay batch-to-batch variation. We included four independent breast tumor samples as technical replicates in each assay plate. The technical replicate breast tumor specimens were identified from Beth Israel Deaconess Medical Center. Sufficient RNA was extracted using the Qiagen AllPrep Kit at a single time point to include in all assay plates. First, we excluded ten specimens (one cel file per specimen) that could not be read by hGlueQC. We normalized the data using the robust median average (RMA) method. Next, we evaluated sample quality and excluded 124 NHS specimens with less than 0.55 area under the curve (AUC) threshold for distinguishing a positive and negative probe signal. Then, we evaluated the association of the first fifty principal components (PCs) in the combined tumor adjacent normal and tumor specimens after robust median average normalization with assay batch. We clipped the first PC associated assay batch

### Adequacy of FFPE-based expression assays

The NHS provides expression data on 326 FFPE samples. To assess the biological interpretability of the NHS expression assays based on FFPE tissue, we obtained the RSEM-based RNA-seq gene quantifications on 1020 non-FFPE samples from the TCGA BRCA cohort. Using HUGO identifiers for genes, the two platforms had 9316 features in common. For platforms p∈[22] we fit (using regularized linear models [38]) the 9316 models

$$V_{igp}\;=\;a_{gp}\;+\;{xi'\beta }_{gp\;}\;+e_{gp}$$

with g indexing genes and i indexing individuals assayed on platform p. Here y_igp_ denotes the normalized expression level for gene g measured on subject i in platform p, x_i_ is a dummy 3-vector indicating whether patient i is +-, -+, or ++ for ER/PR tumor status, and e_igp_ is a homoskedastic error assumed independent across individuals. The (regularized) F statistics for H_o_:β_gp_ = 0 were computed for each platform as a basic measure of assay sensitivity to differences in tumor biology. Owing to the different sample sizes, the denominator degrees of freedom for the F statistics are very different (322 for NHS and 1016 for TCGA). We use the statistics to rank the models, and thereby genes, according to their capacity to distinguish expression variation between samples derived from tumors of different hormone receptor types.

### CADD annotation

Combined Annotation Dependent Depletion (CADD)[11] is a tool for scoring the deleteriousness of SNPs (included in Table 1). CADD integrates multiple annotations into one metric by contrasting variants that survived natural selection with simulated mutations. C-scores are highly associated with allelic diversity, pathogenicity of both coding and non-coding variants and experimentally measured regulatory effects and also highly rank causal variants within individual genome sequences. CADD can quantitatively prioritize functional, deleterious and disease causal variants across a wide range of functional categories, effect sizes, and genetic architectures.

### Statistical analysis

To identify regulatory variants, we investigated the association of 71 breast cancer risk alleles (each entered as a single continuous dosage variable as counts of the minor allele) in linear regression models to identify associations with normalized gene expression levels (eQTL) or biological pathways (fQTL). Covariate information for the breast cancer cases was obtained from biennial questionnaires or extracted from medical records or a supplemental questionnaire. We included patient’s age at diagnosis and year of diagnosis along with array plate identifier as covariates in the multivariate linear regression model, and assumed an additive effect of the SNP on the gene expression measurement. For s = 1,…, 71 enumerating the risk SNP, g = 1,…, 26001 HTA 1.0 transcript clusters, and individuals i contributing tissues of a given type, let *y*_*ig*_ denote the expression level of gene g for subject i, $X_{i}^{t}$ denote the vector of subject's age of diagnosis, year of diagnosis, array plate number, and contribution to the principal component adjustment for expression heterogeneity, and let *D*_*is*_ denote the dosage of the risk allele for SNP s. Statistical significance of the association between SNP s and gene g is determined using the FDR based on the moderated t-statistic for testing the null hypothesis Ho: *β*_2*sg*_ = 0 in the models

$$y_{ig}\;=\;\beta_{0sg}\;+X_{i}^{t}\beta_{1sg}\;+\;\beta_{2sg}D_{is}\;+\epsilon_{isg},$$

where *∈*_*isg*_ denotes a random quantity with mean zero and finite variance $\sigma_{sg}^{2}$. For the analysis of paired tumor-adjacent normal samples, the model is elaborated to include fixed effects of tissue type (dichotomous, tumor or normal) and allelic dose, and the parameter of interest is the coefficient of the product of tissue type code and allelic dose.

Association tests were conducted separately for breast tumor (total tumor n = 376; by estrogen receptor 1 alpha [ERα] by IHC: ER+ tumor n = 262, ER- tumor n = 70) and tumor adjacent normal samples (n = 264) using Affymetrix Power Tools and the Bioconductor package linear models for microarray data (LIMMA)[38]. LIMMA computes modified gene-specific *t*-statistics for general linear predictors of mean expression, and incorporates the Benjamin-Hochberg FDR multiple testing correction technique. Among 250 paired tumor-normal specimens, we also conducted regression analysis testing for an interaction between SNP genotype and tissue type, adjusting for all covariates noted above and including a fixed effect of individual contributing the paired samples.

### fQTL analysis

Functional QTL (fQTL) analysis tests the hypothesis that SNPs influence not only the expression of single genes, but multi-gene processes such as those represented in pathways and annotated in pathway databases such as Reactome[39,40] or the Gene Ontology: classification system. Because we are testing for association between SNPs and groups of genes, fQTL analysis identifies significant trans-associations between sequence variants and biological mechanisms or pathways and treats the measurements of gene expression measures on individual genes as repeated measures on processes. This procedure could be regarded as treating the expression data for all genes in a group as a multivariate response. This solution is related to but not identical to approaches for gene set testing in transcriptomics. The novel question we are attempting to answer is whether there are particular genetic variants that are associated with the expression of a functionally-related group of genes.

The rationale underlying the fQTL model is that a locus or loci within the genome may affect the activity of an intermediary genomic factor, such as a transcription factor, micro RNA, or gene promoter region and that this intermediate element may then affect the expression of a set of genes.[14,22–29] Procedures confronting this generic concern include methods by Wolfringer[41], Goeman's global test [42], Barry's SAFE[43], GSEA[44] and several procedures due to Wu and colleagues [45,46], We defined “pathways” using the GO Molecular Function classifications and then were standardized using the mean and standard deviation values for those genes, which were used to calculate Z-scores. Each pathway is then represented as a vector and the size of this vector depends on the number of samples in the analysis. We construct and fit a linear model of the form:

$$y_{ijk}\;=\;\beta_{0}+\beta_{1}{YrDx}_{k}+\beta_{2}{AgeDx}_{k}+\beta_{3}{SNP:Dosage}_{k}+\;\epsilon_{ijk}$$

where YrDx is the year of diagnosis, AgeDx is age of diagnosis and *i* is the gene set, *j* is for the gene within the gene set and *k* is for the sample. The risk parameter *β*_3_ represents the increase in the mean pathway specific Z-score for each unit increase in the SNP-dosage. It is important to note that this model assumes that genes within a biological class have the same variance, and that, by treating genes within a class as repeated measures on a process, we increase the power of the analysis by reducing the effective number of tests in comparison to a more standard eQTL analysis.

In our fQTL analysis we tested for association between SNPs and the expression of 396 gene sets defined by the Gene Ontology Molecular Function classification for both the 376 tumor and the 263 tumor adjacent normal samples.

### Multiple comparisons

To adjust for multiple comparisons, we used the False Discovery Rate (FDR) for both eQTL and fQTL analyses and present results for FDR < 10%[47]. The eQTL analyses incorporated the Benjamini-Hochberg FDR multiple hypotheses correction technique[38]. For the fQTL analyses, we calculated the FDR through a permutation procedure based on the genotype information[48]. By permuting only the genotype information of the samples, the correlation structure within gene expression and genotype data is preserved.

## Supporting Information

S1 Table Identifiers and genomic contexts of 71 breast cancer risk SNP. Context as determined by Bioconductor TxDb.Hsapiens.UCSC.hg19.knownGene. GTEx version 6 was searched for cis-associated expression in breast mammary tissue.
(DOCX)

S2 Table Tissue source and identifiers for 378 trans-eQTL associations at SNP/tissue-type specific FDR < = 10%. Chromosome locations for SNP as in dbSNP build 144, hg19-compatible addresses. Transcript/gene annotation derived from documentation of the HTA 1.0 array. Fold change, average expression, p-value, FDR, and log odds of differential expression in additive genetic model (denoted "B") all derived from limma version 3.26 (24).
(CSV)

S1 Fig Trans eQTL associations identified in ER+ tissue. (TIF)

S2 Fig Trans eQTL associations identified in comparative analysis of expression in tumor and paired adjacent normal tissue. (TIF)
