# Supplementary material for: Expression Quantitative Trait loci (QTL) in tumor adjacent normal breast tissue and breast tumor tissue
Source: PLoS One. 2017 Feb 2;12(2):e0170181. doi: 10.1371/journal.pone.0170181 (PMC5289428; doi:10.1371/journal.pone.0170181)
Supplement: S1 Table — Context as determined by Bioconductor TxDb.Hsapiens.UCSC.hg19.knownGene. GTEx version 6 was searched for cis-associated expression in breast mammary tissue. (DOCX) [file pone.0170181.s001.docx]

**Supplementary Table 1**. Identifiers and genomic contexts of 71 breast cancer risk SNP. Context as determined by Bioconductor TxDb.Hsapiens.UCSC.hg19.knownGene. GTEx version 6 was searched for cis-associated expression in breast mammary tissue.

| dbSNP id | chr | Position | Context | Entrez ID | Symbol | GTEx_breast_genes  in cis (1Mb) |
| --- | --- | --- | --- | --- | --- | --- |
| rs616488 | chr1 | 10566215 | intron | 5195 | PEX14 |  |
| rs11552449 | chr1 | 114448389 | fiveUTR | 64858 | DCLRE1B |  |
| rs11249433 | chr1 | 121280613 | intron | 647121 | EMBP1 | RP11-439A17.7 |
| rs6678914 | chr1 | 202187176 | intron | 59352 | LGR6 |  |
| rs4245739 | chr1 | 204518842 | threeUTR | 4194 | MDM4 |  |
| rs12710696 | chr2 | 19320803 | intergenic | NA | NA |  |
| rs4849887 | chr2 | 121245122 | intergenic | NA | NA |  |
| rs2016394 | chr2 | 172972971 | intergenic | NA | NA |  |
| rs1550623 | chr2 | 174212894 | intergenic | NA | NA |  |
| rs1045485 | chr2 | 202149589 | coding | 841 | CASP8 |  |
| rs13387042 | chr2 | 217905832 | intergenic | NA | NA |  |
| rs16857609 | chr2 | 218296508 | intergenic | NA | NA |  |
| rs6762644 | chr3 | 4742276 | intron | 3708 | ITPR1 |  |
| rs4973768 | chr3 | 27416013 | intergenic | NA | NA |  |
| rs12493607 | chr3 | 30682939 | intron | 7048 | TGFBR2 |  |
| rs9790517 | chr4 | 106084778 | intron | 54790 | TET2 |  |
| rs6828523 | chr4 | 175846426 | intron | 11086 | ADAM29 |  |
| rs10069690 | chr5 | 1279790 | intergenic | NA | NA |  |
| rs10941679 | chr5 | 44706498 | intergenic | NA | NA |  |
| rs889312 | chr5 | 56031884 | intergenic | NA | NA |  |
| rs1353747 | chr5 | 58337481 | intergenic | NA | NA |  |
| rs1432679 | chr5 | 158244083 | intergenic | NA | NA |  |
| rs11242675 | chr6 | 1318878 | intergenic | NA | NA |  |
| rs204247 | chr6 | 13722523 | intergenic | NA | NA |  |
| rs17529111 | chr6 | 82128386 | intergenic | NA | NA |  |
| rs17530068 | chr6 | 82193109 | intergenic | NA | NA |  |
| rs3757318 | chr6 | 151914113 | intron | 80129 | CCDC170 |  |
| rs12662670 | chr6 | 151918856 | intron | 80129 | CCDC170 |  |
| rs2046210 | chr6 | 151948366 | intergenic | NA | NA |  |
| rs720475 | chr7 | 144074929 | intron | 7984 | ARHGEF5 | ARHGEF34P |
| rs9693444 | chr8 | 29509616 | intergenic | NA | NA |  |
| rs6472903 | chr8 | 76230301 | intergenic | NA | NA |  |
| rs13281615 | chr8 | 128355618 | intergenic | NA | NA |  |
| rs11780156 | chr8 | 129194641 | intergenic | NA | NA |  |
| rs1011970 | chr9 | 22062134 | intron | 100048912 | CDKN2B-AS1 | |
| rs10759243 | chr9 | 110306115 | intergenic | NA | NA |  |
| rs865686 | chr9 | 110888478 | intergenic | NA | NA |  |
| rs2380205 | chr10 | 5886734 | intergenic | NA | NA |  |
| rs11814448 | chr10 | 22315843 | intergenic | NA | NA |  |
| rs10995190 | chr10 | 64278682 | intron | 22891 | ZNF365 |  |
| rs704010 | chr10 | 80841148 | intron | 57178 | ZMIZ1 |  |
| rs7904519 | chr10 | 114773927 | intron | 6934 | TCF7L2 |  |
| rs11199914 | chr10 | 123093901 | intergenic | NA | NA |  |
| rs2981579 | chr10 | 123337335 | intergenic | NA | NA |  |
| rs3817198 | chr11 | 1909006 | intron | 4046 | LSP1 |  |
| rs3903072 | chr11 | 65583066 | intergenic | NA | NA |  |
| rs614367 | chr11 | 69328764 | intergenic | NA | NA |  |
| rs11820646 | chr11 | 129461171 | intergenic | NA | NA |  |
| rs12422552 | chr12 | 14413931 | intergenic | NA | NA |  |
| rs10771399 | chr12 | 28155080 | intergenic | NA | NA |  |
| rs17356907 | chr12 | 96027759 | intergenic | NA | NA |  |
| rs1292011 | chr12 | 115836522 | intergenic | NA | NA |  |
| rs11571833 | chr13 | 32972626 | coding | 675 | BRCA2 |  |
| rs2236007 | chr14 | 37132769 | intron | 5083 | PAX9 |  |
| rs2588809 | chr14 | 68660428 | intron | 5890 | RAD51B |  |
| rs999737 | chr14 | 69034682 | intron | 5890 | RAD51B |  |
| rs941764 | chr14 | 91841069 | intergenic | NA | NA |  |
| rs3803662 | chr16 | 52586341 | intergenic | NA | NA |  |
| rs17817449 | chr16 | 53813367 | intron | 79068 | FTO |  |
| rs11075995 | chr16 | 53855291 | intron | 79068 | FTO |  |
| rs13329835 | chr16 | 80650805 | intergenic | NA | NA |  |
| rs6504950 | chr17 | 53056471 | intron | 252983 | STXBP4 | STXBP4 |
| rs527616 | chr18 | 24337424 | intergenic | NA | NA |  |
| rs8170 | chr19 | 17389704 | coding | 29086 | BABAM1 | ANKLE1(sic) |
| rs8100241 | chr19 | 17392894 | coding | 126549 | ANKLE1 |  |
| rs4808801 | chr19 | 18571141 | intergenic | NA | NA |  |
| rs3760982 | chr19 | 44286513 | intergenic | NA | NA | ZNF404 |
| rs2284378 | chr20 | 32588095 | intron | 22913 | RALY |  |
| rs2823093 | chr21 | 16520832 | intergenic | NA | NA |  |
| rs132390 | chr22 | 29621477 | intron | 129080 | EMID1 |  |
| rs6001930 | chr22 | 40876234 | intergenic | NA | NA |  |
