# Supplementary figures and images for: Expression Quantitative Trait loci (QTL) in tumor adjacent normal breast tissue and breast tumor tissue
Source: PLoS One. 2017 Feb 2;12(2):e0170181. doi: 10.1371/journal.pone.0170181 (PMC5289428; doi:10.1371/journal.pone.0170181)

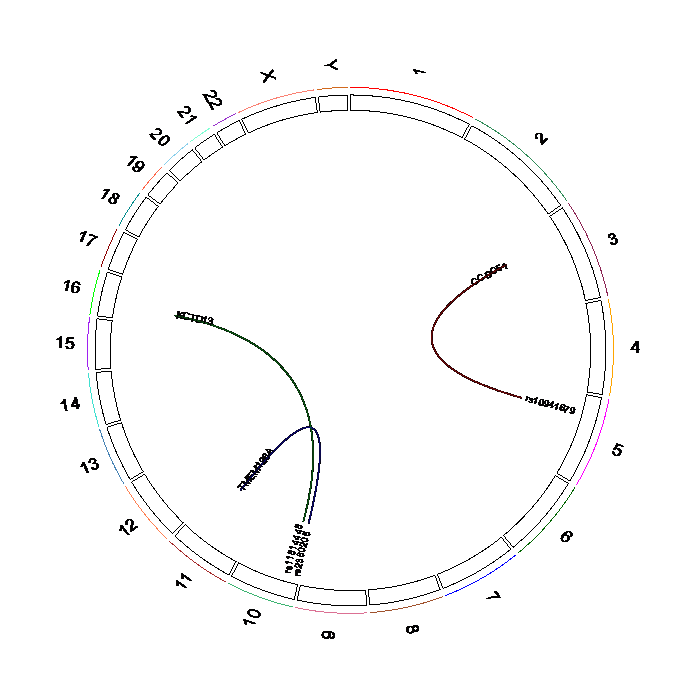

Supplement: S1 Fig — (TIF) [file pone.0170181.s003.tif]

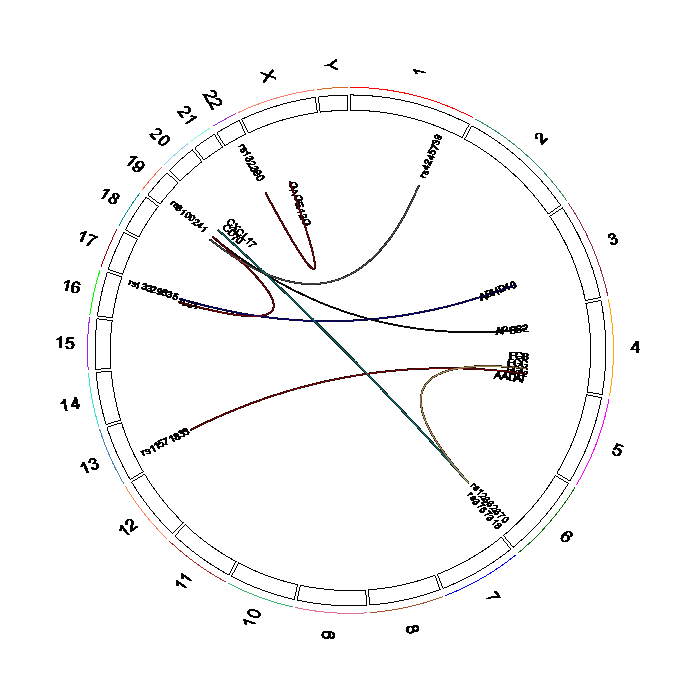

Supplement: S2 Fig — (TIF) [file pone.0170181.s004.tif]
